# Supplementary material for: Extinction and the U.S. Endangered Species Act
Source: PeerJ. 2019 Apr 22;7:e6803. doi: 10.7717/peerj.6803 (PMC6482936; doi:10.7717/peerj.6803)
Supplement: Supplemental Information 1 — Extinct or possibly extinct species broken out by whether last seen before or after protection was enacted, including relevant source data and literature cited. [file peerj-07-6803-s001.pdf]

Table 1. Extinct and possibly extinct species last seen before listing under the Endangered Species Act.

Common Name	Scientific	Listed	Last Seen	Status	NS/IUCN	Taxonomic Group	Region	Source
Oahu tree snail	Achatinella buddii	1981	~1900	Possibly extinct	GX/EX	Mollusk	Hawaii and Pacific I	USFWS 1992, 2011a
Oahu tree snail	Achatinella casta	1981	<1900	Possibly extinct	GX/EX	Mollusk	Hawaii and Pacific I	USFWS 2011a
Oahu tree snail	Achatinella caesia	1981	~1900	Possibly extinct	GX/EX	Mollusk	Hawaii and Pacific I	USFWS 1992, 2011a
Oahu tree snail	Achatinella cestus	1981	1966	Possibly extinct	G1 TH/CR	Mollusk	Hawaii and Pacific I	USFWS 2011a
Oahu tree snail	Achatinella decora	1981	1900	Possibly extinct	GX/EX	Mollusk	Hawaii and Pacific I	USFWS 2011a
Oahu tree snail	Achatinella dimorpha	1981	1967	Possibly extinct	GH/EX	Mollusk	Hawaii and Pacific I	USFWS 2011a
Oahu tree snail	Achatinella elegans	1981	1952	Possibly extinct	G1 TH/EX	Mollusk	Hawaii and Pacific I	USFWS 2011a
Oahu tree snail	Achatinella juddii	1981	1958	Possibly extinct	GH/EX	Mollusk	Hawaii and Pacific I	USFWS 2011a
Oahu tree snail	Achatinella juncea	1981	Unknown	Possibly extinct	GH/EX	Mollusk	Hawaii and Pacific I	USFWS 2011a
Oahu tree snail	Achatinella lehuiensis	1981	1922	Possibly extinct	GX/EX	Mollusk	Hawaii and Pacific I	USFWS 2011a
Oahu tree snail	Achatinella lorata	1981	1979	Possibly extinct	GH/CR	Mollusk	Hawaii and Pacific I	USFWS 2011a
Oahu tree snail	Achatinella papyracea	1981	<1945	Possibly extinct	GX/EX	Mollusk	Hawaii and Pacific I	USFWS 1992, 2011a
Oahu tree snail	Achatinella phaeozona	1981	1974	Possibly extinct	GH/CR	Mollusk	Hawaii and Pacific I	USFWS 1992, 2011a
Oahu tree snail	Achatinella pupukanioe	1981	1980	Possibly extinct	GH/CR	Mollusk	Hawaii and Pacific I	USFWS 2011a
Oahu tree snail	Achatinella rosea	1981	1949	Possibly extinct	G1 TX/EX	Mollusk	Hawaii and Pacific I	USFWS 1992, 2011a
Oahu tree snail	Achatinella spaldingi	1981	1938	Possibly extinct	GX/EX	Mollusk	Hawaii and Pacific I	USFWS 1992, 2011a
Oahu tree snail	Achatinella swiftii	1981	1970's	Possibly extinct	GH/CR	Mollusk	Hawaii and Pacific I	USFWS 1992, 2011a
Oahu tree snail	Achatinella taeniolata	1981	1966	Possibly extinct	GH/CR	Mollusk	Hawaii and Pacific I	USFWS 1992, 2011a
Oahu tree snail	Achatinella thaanumi	1981	1900	Possibly extinct	GX/EX	Mollusk	Hawaii and Pacific I	USFWS 1992, 2011a
Oahu tree snail	Achatinella turgida	1981	1974	Possibly extinct	GX/CR	Mollusk	Hawaii and Pacific I	USFWS 1992, 2011a
Oahu tree snail	Achatinella valida	1981	1951	Possibly extinct	GX/EX	Mollusk	Hawaii and Pacific I	USFWS 1992, 2011a
Oahu tree snail	Achatinella viridans	1981	1979	Possibly extinct	GH/CR	Mollusk	Hawaii and Pacific I	USFWS 1992, 2011a
Oahu tree snail	Achatinella vittata	1981	1953	Possibly extinct	G1 TH/EX	Mollusk	Hawaii and Pacific I	USFWS 1992, 2011a
Oahu tree snail	Achatinella vulpina	1981	1965	Possibly extinct	GH/CR	Mollusk	Hawaii and Pacific I	USFWS 1992, 2011a
Oahu tree snail	Achatinella bellula	1981	1981	Possibly extinct	GH/CR	Mollusk	Hawaii and Pacific I	USFWS 2011a
Kauai ‘akialoa	Akialoa stejnegeri	1967	1965	Possibly extinct	GX/EX	Bird	Hawaii and Pacific I	USFWS 2009
Brown's pigweed	Amaranthus brownii	1996	1983	Possibly extinct	G1/CR	Plant	Hawaii and Pacific I	USFWS 2017a
Culebra Island giant anole	Anolis roosevelti	1977	1932	Possibly extinct	NA/CRPE	Reptile	Southeast	USFWS 2014a
Ivory-billed woodpecker	Campephilus principalis	1967	1944	Possibly extinct	G1/CRPE	Bird	Southeast	USFWS 2010a
Longjaw cisco	Coregonus alpenae	1967	1967	Extinct, delisted	NA/EX	Fish	Midwest	USFFWS 1983a
Haha (Cyanea copelandii ssp. copelandii)	Cyanea copelandii ssp. copelandii	1994	1957	Possibly extinct	G1TH/EX	Plant	Hawaii and Pacific I	USFWS 2012
Long-foot cyanea	Cyanea dolichopoda	2010	1990	Possibly extinct	GH/EX	Plant	Hawaii and Pacific I	USFWS 2017b
'Oha (Cyanea eleeleensis)	Cyanea eleeleensis	2010	1977	Possibly extinct	GH/EX	Plant	Hawaii and Pacific I	USFWS 2017c
Haha	Cyanea kolekoleensis	2010	1998	Possibly extinct	G1/CRPE	Plant	Hawaii and Pacific I	USFWS 2017d
Haha	Cyanea kuhihewa	2010	2001	Possibly extinct	GH/CRPE	Plant	Hawaii and Pacific I	USFWS 2017e
Maui cyanea	Cyanea mauiensis	2013	1918	Possibly extinct	GH/EX	Plant	Hawaii and Pacific I	USFWS 2016a
No common name	Cyperus neokunthianus	2016	1996	Possibly extinct	GH/NA	Plant	Hawaii and Pacific I	USFWS 2016a
Tecopa pupfish	Cyprinodon nevadensis calidae	1970	1970	Extinct, delisted	G2TX/NA	Fish	Pacific Southwest	USFWS 1982, Miller et al. 1989
Ha`iwale	Cyrtandra crenata	1994	1947	Possibly extinct	GH/CRPE	Plant	Hawaii and Pacific I	USFWS 2011b
Waiolani Cyrtandra	Cyrtandra waiolani	2012	2005	Possibly extinct	GH/EX	Plant	Hawaii and Pacific I	USFWS 2012b
Opuhe nalo	Drosophila aglaia	2006	1997	Possibly extinct	G1/NA	Arthropod	Hawaii and Pacific I	USFWS 2012c, Magnacca 2018
Picture-wing fly	Drosophila differens	2006	1999	Possibly extinct	G1/NA	Arthropod	Hawaii and Pacific I	USFWS 2018a, Magnacca 2018
Nalo mea hula	Drosophila neoclavisetae	2006	1975	Possibly extinct	G1/NA	Arthropod	Hawaii and Pacific I	USFWS 2018b, Magnacca 2018
Picture-wing fly	Drosophila sharpi	2006	1991	Possibly extinct	G1/NA	Arthropod	Hawaii and Pacific I	USFWS 2017f, Magnacca 2018
Mull's picture-wing	Drosophila tarphytrichia	2006	1997	Possibly extinct	G1/NA	Arthropod	Hawaii and Pacific I	USFWS 2012d, Magnacca 2018
Na‘ena‘e	Dubautia kenwoodii	2010	1991	Possibly extinct	G1/CRPE	Plant	Hawaii and Pacific I	USFWS 2017g

Yellow-blossom pearly mussel	Epioblasma florentina florentina	1976	1967	Extinct, delisting recommended	G1TX/EX	Mollusk	Southeast	USFWS 2017h
Upland combshell	Epioblasma metastriata	1993	1988	Extinct, delisting recommended	GH/CR	Mollusk	Southeast	USFWS 2008
Southern acornshell	Epioblasma othcaloogensis	1993	1974	Extinct, delisting recommended	GHQ/CR	Mollusk	Southeast	USFWS 2008
Sampson's pearlymussel	Epioblasma sampsonii	1976	1935	Extinct, delisted	GX/EX	Mollusk	Midwest	USFWS 1984
Tubercled blossom	Epioblasma torulosa torulosa	1976	1969	Extinct, delisting recommended	G2TX/EX	Mollusk	Southeast	USFWS 2017h
Turgid blossom pearly mussel	Epioblasma turgidula	1976	1965	Extinct, delisting recommended	GX/EX	Mollusk	Southeast	USFWS 2017h
Amistad gambusia	Gambusia amistadensis	1980	1974	Extinct, delisted	GX/EX	Fish	Southwest	USFWS 1987, Miller et al. 1989
Maui nukupu‘u	Hemignathus affinis	1970	1967	Possibly extinct	G1T1/CRP	Bird	Hawaii and Pacific I	USFWS 2006, USFWS 2010 b
Kauai nukupu‘u	Hemignathus hanapepe	1967	ıgs in 1995	Possibly extinct	G1T1/CRP	Bird	Hawaii and Pacific I	USFWS 2006, USFWS 2010b
Santa Barbara song sparrow	Melospiza melodia graminea	1973	1959	Extinct, delisted	G5T1/NA	Bird	Pacific Southwest	USFWS 1983b
West Indian monk seal	Monachus tropicalis	1979	1952	Extinct, delisted	GX/EX	Mammal	Southeast	NMFS 2008
Guam broadbill	Myiagra freycineti	1984	1984	Extinct, delisted	NA/EX	Bird	Hawaii and Pacific I	USFWS 2004
Scioto madtom	Noturus trautmani	1975	1957	Extinct, delisting recommended	GX/EX	Fish	Midwest	USFWS 2009b
Eskimo curlew	Numenius borealis	1967	1963	Possibly extinct	GH/EX	Bird	Alaska	USFWS 2016b
Holei	Ochrosia kilaueaensis	1994	1927	Possibly extinct	GH/CR	Plant	Hawaii and Pacific I	USFWS 2012e
Squirrel Chimney Cave Shrimp	Palaemonetes cummingi	1990	1973	Possibly extinct	GH/CR	Arthropod	Southeast	USFWS 2016c
Molokai creeper	Paroreomyza flammea	1970	1963	Possibly extinct	GH/EX	Bird	Hawaii and Pacific I	USFWS 2015a
Phyllostegia glabra var. lanaiensis	Phyllostegia glabra var. lanaiensis	1991	1914	Possibly extinct	G3TH/NA	Plant	Hawaii and Pacific I	USFWS 2012f
Flat pigtoe	Pleurobema marshalli	1987	1980	Extinct, delisting recommended	GX/CR	Mollusk	Southeast	USFWS 2015b
Little Mariana fruit bat	Pteropus tokudae	1984	1968	Extinct, delisting recommended	NA	Mammal	Hawaii and Pacific I	USFWS 2015c
Eastern cougar	Puma concolor couguar	1973	1938	Extinct, delisted	G5TXQ/CI	Mammal	Northeast	USFWS 2018c
Stirrup shell	Quadrula stapes	1987	1984	Extinct, delisting recommended	GH/CR	Mollusk	Southeast	USFWS 2015b
Blue pike	Sander vitreus glaucus	1967	1965	Extinct, delisted	G5TX/NA	Fish	Midwest	USFWS 1983a, Miller et al. 1989
Bachman's warbler	Vermivora bachmanii	1967	1962	Possibly extinct	GH/CR	Bird	Southeast	USFWS 2015d
Guam bridled white-eye	Zosterops conspicillatus conspicill	1984	1983	Extinct, delisting recommended	NA/EX	Bird	Hawaii and Pacific I	USFWS 2015

Literature Cited:

Miller, R.R., Williams, J.D. & Williams, J.E., (1989). Extinctions of North American fishes during the past century. Fisheries 14(6):22-38.

National Marine Fisheries Service, (2008). Federal Register, Endangered and Threatened Species; Final Rule to Remove the Caribbean Monk Seal From the Federal List of Endangered and Threatened Wildlife. https://www.gpo.gov/fdsys/pkg/FR-2008-10-28/pdf/E8-25704.pdf#page

U.S. Fish and Wildlife Service, (1982). Deregulation of the Tecopa Pupfish. Federal Register, Vol. 47, No 10: 2317-2319. https://ecos.fws.gov/docs/federal_register/fr572.pdf

U.S. Fish and Wildlife Service, (1983a). Deregulation of the Longjaw Cisco and the Blue Pike. September 2, 1983. Federal Register, Vol. 48, No. 172: 39941-39943. https://ecos.fws.gov/docs/federal_register/fr735.pdf

U.S. Fish and Wildlife Service, (1983b). Removal of the Santa Barbara Song Sparrow from the List of Endangered Species. Federal Register, Vol. 48, No. 198: 46336-46337. https://ecos.fws.gov/docs/federal_register/fr754.pdf

U.S. Fish and Wildlife Service, (1984). Removal of Epioblasma sampsoni, Sampson's pearly Mussel, from the list of Endangered and Threatened wildlife. Federal Register, Vol. 49, No.5: 1057-1058. https://ecos.fws.gov/docs/federal_register/fr781.pdf

U.S. Fish and Wildlife Service, (1987). Removal of gambusia amistadensis, the Amistad Gambusia, From the list of Endangered and Threatened Wildlife. Federal Register, Vol.52, No 233: 46083-46087. https://ecos.fws.gov/docs/federal_register/fr1362.pdf

U.S. Fish and Wildlife Service, (1992). Recovery Plan for the O'ahu tree snails of the genus Achatinella. https://esadocs.cci-dev.org/ESAdocs/recovery_plan/920630.pdf

U.S. Fish and Wildlife Service, (2004). Removing the Mariana Mallard and the Guam broadbill from the Federal List of Endangered and Threatened Wildlife. Federal Register, Vol. 69, No. 35: 8116-8119. https://www.gpo.gov/fdsys/pkg/FR-2004-02-23/pdf/04-3784.pdf#page=1

U.S. Fish and Wildlife Service, (2006). Revised Recovery Plan for Hawaiian Forest Birds. https://ecos.fws.gov/docs/recovery_plans/2006/060922a_docs/doc760.pdf

U.S. Fish and Wildlife Service, (2008). 5-Year Review, Summary and Evaluation, Upland Combshell (Epioblasma metastriata), Southern acornshell (Epioblasma othcaloogensis) https://ecos.fws.gov/docs/five_year_review/doc2365.pdf

U.S. Fish and Wildlife Service, (2009a). 5-year Review, Summary and Evaluation, Kaua'i'akialoa (Hemignathus procerus, now Akialoa stejnegeri). https://ecos.fws.gov/docs/five_year_review/doc2533.pdf

U.S. Fish and Wildlife Service, (2009b). 5-Year Review, Summary and Evaluation, Scioto Madtom (Noturus trautmani). https://ecos.fws.gov/docs/five_year_review/doc3057.pdf
U.S. Fish and Wildlife Service, (2010a). Recovery Plan for the Ivory-billed Woodpecker (Campephilus principalis). U.S. Fish and Wildlife Service. http://ecos.fws.gov/docs/recovery_plan/100719.pdf

U.S. Fish and Wildlife Service, (2010b). 5-Year Review: Summary and Evaluation, Kaua'i nukupu'u (Hemignathus lucidus hanapepe) and Maui nukupu'u (Hemignathus lucidus affinis). https://ecos.fws.gov/docs/five_year_review/doc3858.pdf

U.S. Fish and Wildlife Service, (2011a). Oahu tree snails (Achatinella spp.) 5-Year Review. https://ecos.fws.gov/docs/five_year_review/doc3903.pdf

U.S. Fish and Wildlife Service, (2011b). 5-Year Review: Short Form Summary, Cyrtandra crenata (ha'iwale). https://ecos.fws.gov/docs/five_year_review/doc3819.pdf

U.S. Fish and Wildlife Service, (2012a). 5-Year Review: Summary and Evaluation, Cyanea copelandii copelandii (Haha). https://ecos.fws.gov/docs/five_year_review/doc4088.pdf

U.S. Fish and Wildlife Service, (2012b). Federal Register. Endangered and Threatened Wildlife and Plants; Endangered Status for 23 Species on Oahu and Designation of Critical Habitat for 124 Species; Final Rule. Vol. 77 No.181: 57653. http://www.gpo.gov/fdsys/pkg/FR-2012-09-18/pdf/2012-19561.pdf

U.S. Fish and Wildlife Service, (2012c). 5-Year Review: Summary and Evaluation, picture-wing fly (Drosophila aglaia). https://ecos.fws.gov/docs/five_year_review/doc4103.pdf

U.S. Fish and Wildlife Service, (2012d). 5-Year Review: Summary and Evaluation, Picture-wing fly (Drosophila tarphytrichia). https://ecos.fws.gov/docs/five_year_review/doc4092.pdf

U.S. Fish and Wildlife Service, (2012e). 5-Year Review: Summary and Evaluation. Ochrosia Kilaueaensis (Holei). https://ecos.fws.gov/docs/five_year_review/doc4071.pdf

U.S. Fish and Wildlife Service, (2012f). 5-Year Review. Phyllostegia glabra var. lanaiensis (No common name). https://ecos.fws.gov/docs/five_year_review/doc4068.pdf

U.S. Fish and Wildlife Service, (2015a). 5-Year Review: Short Form Summary, Kakawahie or Molokai creeper (Paroreomyza flammea). https://ecos.fws.gov/docs/five_year_review/doc4575.pdf

U.S. Fish and Wildlife Service, (2015b). 5-Year Review: Summary and Evaluation, Black Clubshell (Curtus' Pearly Mussel), Stirrup shell (Quadrula stapes), Flat Pigtoe (Marshall's Pearly Mussel)(Pleurobema marshalli). https://ecos.fws.gov/docs/five_year_review/doc4642.pdf

U.S. Fish and Wildlife Service, (2015c). 5-Year Review: Short Form Summary, Pteropus tokudae (little Mariana fruit bat) https://ecos.fws.gov/docs/five_year_review/doc4544.pdf

U.S. Fish and Wildlife Service, (2015d). 5-Year Review, Summary and Evaluation, Bachman's Warbler (Vermivora bachmanii). https://ecos.fws.gov/docs/five_year_review/doc4548.pdf
U.S. Fish and Wildlife Service, (2015e). 5-Year Review: Short Form Summary, Zosterops conipicillatus conspicillatus (Guam bridled white-eye) https://ecos.fws.gov/docs/five_year_review/doc4545.pdf

U.S. Fish and Wildlife Service, (2016a). Endangered status for 49 species from the Hawaiian Islands. Federal Register, Vol. 81, No. 190: 67786 -67860. https://www.govinfo.gov/content/pkg/FR-2016-09-30/pdf/2016-23112.pdf

U.S. Fish and Wildlife Service, (2016b). 5-Year Review: Summary and Evaluation, Eskimo Curlew (Numenius borealis). https://ecos.fws.gov/docs/five_year_review/doc4866.pdf

U.S. Fish and Wildlife Service. (2016c). 5-Yeaer Review: Summary and Evaluation , Squirrel Chimney Cave Shrimp (Palaemonetes cummingi). https://ecos.fws.gov/docs/five_year_review/doc5095.pdf

U.S. Fish and Wildlife Service, (2017a). 5-year review, short form summary, Amaranthus brownii (No common name). https://ecos.fws.gov/docs/five_year_review/doc5238.pdf

U.S. Fish and Wildlife Service, (2017b). 5-Year Review, Cyanea dolichopoda. https://ecos.fws.gov/docs/five_year_review/doc5247.pdf

U.S. Fish and Wildlife Service, (2017c). 5-Year Review: summary and evaluation, Cyanea eleeleensis. https://ecos.fws.gov/docs/five_year_review/doc5248.pdf

U.S. Fish and Wildlife Service, (2017d). 5-Year Review: summary and evaluation, Cyanea Kolekoleensis. https://ecos.fws.gov/docs/five_year_review/doc5249.pdf

U.S. Fish and Wildlife Service, (2017e). 5-Year Review: summary and evaluation, Cyanea kuhihewa. https://ecos.fws.gov/docs/five_year_review/doc5250.pdf

U.S. Fish and Wildlife Service, (2017f). 5-Year Review: summary and evaluation, picture-wing fly (Drosophilla sharpi). https://ecos.fws.gov/docs/five_year_review/doc5232.pdf

U.S. Fish and Wildlife Service, (2017g). 5-Year Review: summary and evaluation, Dubautia Kenwoodii (Na'ena'e). https://ecos.fws.gov/docs/five_year_review/doc5264.pdf

U.S. Fish and Wildlife Service, (2017h). 5-Year Review: Summary and Evaluation, Green-Blossom pearly mussel (Epioblasma torulosa gubernaculum), Tubercled-blossom pearly mussel (Epioblasma torulosa torulosa), Turgid-blossom pearly mussel (Epioblasma turgidula), Yellow-Blossom pearly mussel (Epioblasma florentina florentina). https://ecos.fws.gov/docs/five_year_review/doc5656.pdf

U.S. Fish and Wildlife Service, (2018a). 5-Year Review: Summary and Evaluation, picture-wing fly (Drosophila differens). https://ecos.fws.gov/docs/five_year_review/doc4102.pdf

U.S. Fish and Wildlife Service, (2018b). 5-Year Review: Summary and Evaluation, picture-wing fly (Drosophila neoclavisetae) https://ecos.fws.gov/docs/five_year_review/doc4096.pdf

U.S. Fish and Wildlife Service, (2018c). Federal Register, Endangered and Threatened Wildlife and Plants; Removing the Eastern Puma (=cougar) From the Federal List of Endangered and Threatened Wildlife. https://www.gpo.gov/fdsys/pkg/FR-2018-01-23/pdf/2018-01127.pdf

Table 2. Extinct or possibly extinct species last seen after listing under the Endangered Species Act.

Common Name	Scientific	Listed	Last Seen	Status	NS/IUCN	Taxonomic Group	Region	Abundance at Listing	Reference
Liliwai	Acaena exigua	1992	2000	Possibly extinct	G1/EX	Plant	Hawaii and Pacific Islands	None at listing, one 1997	USFWS, 2014a; USFWS, 2016a
Oahu tree snail curta	Achatinella curta	1981	1989	Possibly extinct	G1/CR	Mollusk	Hawaii and Pacific Islands	Six snails seen 1983	U.S. Army, 2004; USFWS, 2011
Oahu tree snail leucorraphe	Achatinella leucorraphe	1981	1989	Possibly extinct	G1T1/CR	Mollusk	Hawaii and Pacific Islands	Unknown	USFWS, 2011a
Oahu tree snail pulcherrima	Achatinella pulcherrima	1981	1993	Possibly extinct	G1T1/CR	Mollusk	Hawaii and Pacific Islands	<50	USFWS, 2011a
Dusky seaside sparrow	Ammodramus maritimus nigrescens	1967	1987	Extinct, Delisted	G4TX/EX	Bird	Southeast	927 singing males	USFWS 1990
Mariana mallard	Anas oustaleti	1977	1981	Extinct , Delisted	NA	Bird	Hawaii and Pacific Islands	Two males and a female	USFWS 2004
Morro Bay kangaroo rat	Dipodomys heermanni morroensis	1970	1993	Possibly extinct	G3TH/CR	Mammal	Pacific Southwest	>2,000	USFWS 2011b
Fresno kangaroo rat	Dipodomys nitratoides exilis	1985	1992	Possibly extinct	G3TH/NA	Mammal	Pacific Southwest	Unknown	USFWS 2010a
Golden coqui	Eleutherodactylus jasperi	1977	1981	Possibly extinct	NA/CR	Amphibian	Southeast	Unknown	USFWS 2013a
Curtis' pearlymussel	Epioblasma florentina curtisii	1976	1993	Possibly extinct	G1T1	Mollusk	Southeast	>100	USFWS 2010b
White cat's paw pearly mussel	Epioblasma obliquata perobliqua	1976	1999	Possibly extinct	G1T1	Mollusk	Midwest	Two in 1975	USFWS 2013b
Green blossom pearly mussel	Epioblasma torulosa gubernaculum	1976	1982	Extinct, delisting recommended	G2TX/EX	Mollusk	Southeast	One after listing	USFWS 2017a
Slender chub	Erimystax cahni	1977	1996	Possibly extinct	G1/EN	Fish	Southeast	<30	USFWS 2014b
Maryland darter	Etheostoma sellare	1967	1988	Possibly extinct	GH/EX	Fish	Northeast	<50	Burkhead 2012, USFWS 2007
San Marcos gambusia	Gambusia georgei	1980	1982	Extinct	GX/EX	Fish	Southwest	Three	Burkhead 2012, USFWS 1996
Hau kuahiwi	Hibiscadelphus woodii	1996	2006	Possibly extinct	G1/EX	Plant	Hawaii and Pacific Islands	Four trees	USFWS 2017b
Maui ʻākepa	Loxops ochraceus	1970	1988	Possibly extinct	G1TH/CRPE	Plant	Hawaii and Pacific Islands	Unknown	Scott et al. 2008, USFWS 2015a
Poʻouli	Melamprosops phaeosoma	1975	2004	Possibly extinct	G1/CR	Bird	Hawaii and Pacific Islands	Unknown	USFWS 2015b
Kauai ʻoʻo	Moho braccatus	1967	1987	Possibly extinct	GH/EX	Bird	Hawaii and Pacific Islands	Unknown	USFWS 2009a
Molokai thrush	Myadestes lanaiensis rutha	1970	1980	Possibly extinct	GHTH/CRPE	Bird	Hawaii and Pacific Islands	Estimated 36 birds	Scott et al. 2008, USFWS 2018
Large Kauai thrush	Myadestes myadestinus	1970	1987	Possibly extinct	GH/EX	Bird	Hawaii and Pacific Islands	Estimated 337 birds	USFWS 2009b
Oahu creeper	Paroreomyza maculata	1970	1978	Possibly extinct	GH/CRPE	Bird	Hawaii and Pacific Islands	<10	USFWS 2013c
Lotis blue butterfly	Lycaeides argyrognomon lotis	1976	1983	Possibly extinct	G5TH/NA	Arthropod	Pacific Southwest	<30	USFWS 2011c
Black clubshell	Pleurobema curtum	1987	1990	Possibly extinct	GH/CR	Mollusk	Southeast	Unknown	USFWS 2015c
ʻO ʻu	Psittirostra psittacea	1967	1989	Possibly extinct	G1/CRPE	Bird	Hawaii and Pacific Islands	<500	Scott et al. 2008, USFWS 2015d
Pamakani	Tetramolopium capillare	1994	2001	Possibly extinct	G1/NA	Plant	Hawaii and Pacific Islands	<200	USFWS 2012

Literature Cited:

Burkhead, N.M., (2012). Extinction Rates in North American Freshwater Fishes, 1900–2010. *BioScience, 62*, 798-808.

Scott, J. M., Ramsey, F. L., Lammertink, M., Rosenberg, K. V., Rohrbaugh, R., Wiens, J. A., & Reed, J. M., (2008). When is an "extinct" species really extinct? Gauging the search efforts for Hawaiian forest birds and the Ivory-billed Woodpecker. *Avian Conservation and Ecology, 3*, 3.

U.S. Army, (2004). Garrison Hawaii O'ahu Training Areas Natural Resource Management. Final Report. (Achatinella curta). Prepared by the Pacific Cooperative Studies Unit. http://manoa.hawaii.edu/hpicesu/DPW/2004_YER/YER2004_edited.pdf

U.S. Fish and Wildlife Service, (1990). Final Rule to Delist the Dusky Seaside Sparrow and Remove its Critical Habitat Designation. Federal Register, Vol. 55, No. 239: 51112-51114. https://ecos.fws.gov/docs/federal_register/fr1798.pdf

U.S. Fish and Wildlife Service, (1996). Recovery Plan for the San Marcos and Comal Springs and Associated Aquatic Ecosystems (Revised). https://ecos.fws.gov/docs/recovery_plan/960214.pdf

U.S. Fish and Wildlife Service, (2004). Removing the Mariana Mallard and the Guam broadbill from the Federal List of Endangered and Threatened Wildlife. Federal Register, Vol. 69, No. 35: 8116-8119. https://www.gpo.gov/fdsys/pkg/FR-2004-02-23/pdf/04-3784.pdf#page=1

U.S. Fish and Wildlife Service, (2007). 5-Year Review: Summary and Evaluation, Maryland darter (Etheostoma sellare). https://ecos.fws.gov/docs/five_year_review/doc1173.pdf

U.S. Fish and Wildlife Service, (2009a). 5-Year Review, Summary and Evaluation, Kaua'i'o'o (Moho braccatus). https://ecos.fws.gov/docs/five_year_review/doc2534.pdf

U.S. Fish and Wildlife Service, (2009b). 5-Year Review, Summary and Evaluation, Kama'o or Large Kauna'i thrush (Myadestes myadestinus). https://ecos.fws.gov/docs/five_year_review/doc2531.pdf

U.S. Fish and Wildlife Service (2010a), 5-Year Review: Summary and Evaluation, Fresno Kangaroo Rat (Dipodomys nitratodes exilis). https://ecos.fws.gov/docs/five_year_review/doc3214.pdf

U.S. Fish and Wildlife Service, (2010b). 5-Year Review The Curtis' Pearlymussel (Epioblasma florentina curtisii). https://ecos.fws.gov/docs/five_year_review/doc3119.pdf

U.S. Fish and Wildlife Service, (2011a). Oahu tree snails (Achatinella spp.) 5-Year Review. https://ecos.fws.gov/docs/five_year_review/doc3903.pdf

U.S. Fish and Wildlife Service, (2011b). Morro Bay Kangaroo Rat (Dipodomys heermanni morroensis), 5-Year Review: Summary and Evaluation. https://ecos.fws.gov/docs/five_year_review/doc3641.pdf

U.S. Fish and Wildlife Service, (2011c). 5-year Review: Summary and Evaluation, lotis blue butterfly (Lycaeides argyrognomon lotis) https://ecos.fws.gov/docs/five_year_review/doc3960.pdf

U.S. Fish and Wildlife Service, (2012). 5-year Review: Summary and Evaluation, Tetramolopium capillare (Pamakani). https://ecos.fws.gov/docs/five_year_review/doc4056.pdf

U.S. Fish and Wildlife Service, (2013a). 5-Year Review Golden Coqui (Eleutherdodactylus jasperi) https://ecos.fws.gov/docs/five_year_review/doc4276.pdf

U.S. Fish and Wildlife Service, (2013b). 5-Year Review, White Cat's Paw Pearly Mussel (Epioblasma obliquata perobliqua). https://ecos.fws.gov/docs/five_year_review/doc4135.pdf

U.S. Fish and Wildlife Service, (2013c). 5-Year Review: Short Form Summary, Paroreomyza maculate (O'ahu creeper). https://ecos.fws.gov/docs/five_year_review/doc4246.pdf

U.S. Fish and Wildlife Service, (2014a). 5-Year Review, Acaena exigua (Liliwai). https://ecos.fws.gov/docs/five_year_review/doc4384.pdf

U.S. Fish and Wildlife Service, (2014b). 5-Year Review: Slender Chub (Erimystax (=Hybopsis)cahni) https://ecos.fws.gov/docs/five_year_review/doc4357.pdf

U.S. Fish and Wildlife Service, (2015a). 5-Year Review: Short Form Summary, Maui akepa (Loxops coccineus ochraceus) https://ecos.fws.gov/docs/five_year_review/doc4568.pdf

U.S. Fish and Wildlife Service, (2015b). 5-Year Review: Short Form Summary, Po'ouli (Melamprosops Phaeosoma). https://ecos.fws.gov/docs/five_year_review/doc4578.pdf

U.S. Fish and Wildlife Service, (2015c). 5-Year Review: Summary and Evaluation, Black Clubshell (Curtus' Pearly Mussel), Stirrup shell (Quadrula stapes), Flat Pigtoe (Marshall's Pearly Mussel)(Pleurobema marshalli). https://ecos.fws.gov/docs/five_year_review/doc4642.pdf

U.S. Fish and Wildlife Service, (2015d). 5-Year Review: Short Form Summary. Ou (Psittirostra Psittacea) https://ecos.fws.gov/docs/five_year_review/doc4577.pdf

U.S. Fish and Wildlife Service, (2016a). Endangered and threatened wildlife and plants, designation and nondesignation of critical habitat on Molokai, Lanai, Maui, and Kahoolawe for 135 species. Federal Register, 81, 17790-18108.

U.S. Fish and Wildlife Service, (2017a). 5-Year Review: Summary and Evaluation, Green-Blossom pearly mussel (Epioblasma torulosa gubernaculum), Tubercled-blossom pearly mussel (Epioblasma torulosa torulosa), Turgid-blossom pearly mussel (Epioblasma turgidula), Yellow-

Blossom pearly mussel (Epioblasma florentina florentina).
https://ecos.fws.gov/docs/five_year_review/doc5656.pdf

U.S. Fish and Wildlife Service, (2017b). 5-Year Review: summary and evaluation, Dubautia Kenwoodii (Na'ena'e). https://ecos.fws.gov/docs/five_year_review/doc5264.pdf

U.S. Fish and Wildlife Service, (2018). 5-Year Review: Short Form Summary, Olomao or Molokai Thrush (Myadestes lanaiensis rutha.
https://ecos.fws.gov/docs/five_year_review/doc5760.pdf
